# Mitochondrial Oxidative Stress due to Complex I Dysfunction Promotes Fibroblast Activation and Melanoma Cell Invasiveness

**DOI:** 10.1155/2012/684592

**Published:** 2012-01-04

**Authors:** Maria Letizia Taddei, Elisa Giannoni, Giovanni Raugei, Salvatore Scacco, Anna Maria Sardanelli, Sergio Papa, Paola Chiarugi

**Affiliations:** ^1^Department of Biochemical Sciences, Tuscany Tumor Institute, University of Florence, Morgagni Avenue 50, 50134 Florence, Italy; ^2^Center for Research, Transfer and High Education Study at Molecular and Clinical Level of Chronic, Inflammatory, Degenerative and Neoplastic Disorders for the Development on Novel Therapies, University of Florence, 50134 Florence, Italy; ^3^Department of Medical Biochemistry, Biology and Physics, University of Bari, Policlinico, G. Cesare Square 70124 Bari, Italy; ^4^Institute of Biomembrane and Bioenergetic, CNR, Amendola Street 176, 70126 Bari, Italy

## Abstract

Increased ROS (cellular reactive oxygen species) are characteristic of both fibrosis and tumour development. ROS induce the trans-differentiation to myofibroblasts, the activated form of fibroblasts able to promote cancer progression. Here, we report the role of ROS produced in response to dysfunctions of mitochondrial complex I, in fibroblast activation and in tumour progression. We studied human fibroblasts with mitochondrial dysfunctions of complex I, leading to hyperproduction of ROS. We demonstrated that ROS level produced by the mutated fibroblasts correlates with their activation. The increase of ROS in these cells provides a greater ability to remodel the extracellular matrix leading to an increased motility and invasiveness. Furthermore, we evidentiated that in hypoxic conditions these fibroblasts cause HIF-1*α* stabilization and promote a proinvasive phenotype of human melanoma cells through secretion of cytokines. These data suggest a possible role of deregulated mitochondrial ROS production in fibrosis evolution as well as in cancer progression and invasion.

## 1. Introduction

Mitochondrial-produced ROS have been recently involved in metastatic dissemination of cancer cells, as shown by Ishikawa et al. These authors described how replacing the endogenous mitochondrial DNA in a weakly metastatic tumour cell line with mitochondrial DNA from a highly metastatic cell line enhanced tumour progression through increased production of ROS and HIF-1*α* stabilization [1].

Recent studies demonstrate that tumour growth does not depend only on malignant cancer cells themselves but also on the surrounding tumour stroma. Indeed, tumour progression, growth, and spread is strictly dependent on angiogenesis and on cytokines and growth factors secreted by microenvironmental cells [2]. In this context, evidence is increasing that CAFs (cancer-associated fibroblasts) are key determinants in the malignant progression of cancer [3]. These fibroblasts, also commonly referred to as myofibroblasts, are the differentiated form of fibroblast that have acquired contractile and secretory characteristics [4]. They have been initially identified during wound healing [5], but are also present in the reactive tumour stroma, promoting tumour growth and progression [6]. Their role is linked to extracellular matrix deposition and secretion of MMPs (matrix metalloproteinases). Furthermore, activated fibroblasts influence cancer cells through the secretion of growth factors and are able to mediate EMT (epithelial mesenchymal transition) and stemness of tumor cells themselves, supporting their progression and the metastatic process. Transdifferentiation to myofibroblast is dependent on both exposure to MMPs and increased level of cellular ROS [7, 8]. Increased cellular ROS are characteristic of both fibrosis and malignancy. We have recently demonstrated that CAFs induce EMT of prostate cancer cells through a proinflammatory pathway involving COX-2 (cycloxygenase-2), NF-*κ*B (nuclear factor-*κ*B), and HIF-1*α* [9]. The secretion of MMPs by CAFs induces a release of ROS in prostate carcinoma cells, which is mandatory for EMT, stemness, and dissemination of metastatic cells.

The aim of the present work is to assess the role of ROS produced in response to mitochondrial dysfunctions in fibroblast activation and in tumour progression.

Analysis of human fibroblasts with genetic dysfunctions of mitochondrial complex I show that ROS level produced by these fibroblasts correlate with their activation, leading to enhanced motility and invasiveness. Furthermore, in hypoxic conditions, we evidentiated that ROS generated by mitochondrial mutations promote a proinvasive phenotype of melanoma cells though HIF-1*α* stabilization and growth factor secretion.

## 2. Results

### 2.1. ROS Produced by Fibroblasts Carrying Mitochondrial Disfunctions Induce Transdifferentiation to Myofibroblasts

Our interest is to assess the role of mitochondrial oxidative stress for stromal fibroblast activation during tumour progression. To this end we used human fibroblasts carrying mitochondrial dysfunctions of complex I. In particular, fibroblasts mutated in the nuclear *NDUFS1* gene encoding for the 75 kDa-FeS protein (NDUFS1 Q522K and NDUFS1 R557X/T595A) of mitochondrial complex I, fibroblasts mutated in the nuclear *NDUFS4* gene encoding for the 18 kDa subunit (NDUFS4 W15X) of mitochondrial complex I, and fibroblasts mutated in the nuclear *PINK1* gene encoding for the PTEN induced Ser/Thr putative kinase1 localized in mitochondria (PINK W437X), in the same patient this mutation coexists with two homoplasmic mtDNA missense mutations in the *ND5* and *ND6 *genes coding for two subunits of complex I [10, 11]. As control we used neonatal human dermal fibroblasts (HFY). Previously, it has been shown that mutation in *NDUSF4* gene results in complete suppression of the NADH-ubiquinone oxidoreductase activity of complex I, without any ROS accumulation [12]. The Q522K mutation in the *NDUFS1* gene results in a marked, but not complete, suppression of complex I activity with large accumulation of H_2_O_2_ and intramitochondrial superoxide ion [12]. Furthermore, it has been shown that the coexistence of the ND5 and ND6 mutations with the PINK1 mutation, contributes to enhanced ROS production by complex I and to a decrease in the Km for NADH [11, 13].

**Figure 1:**
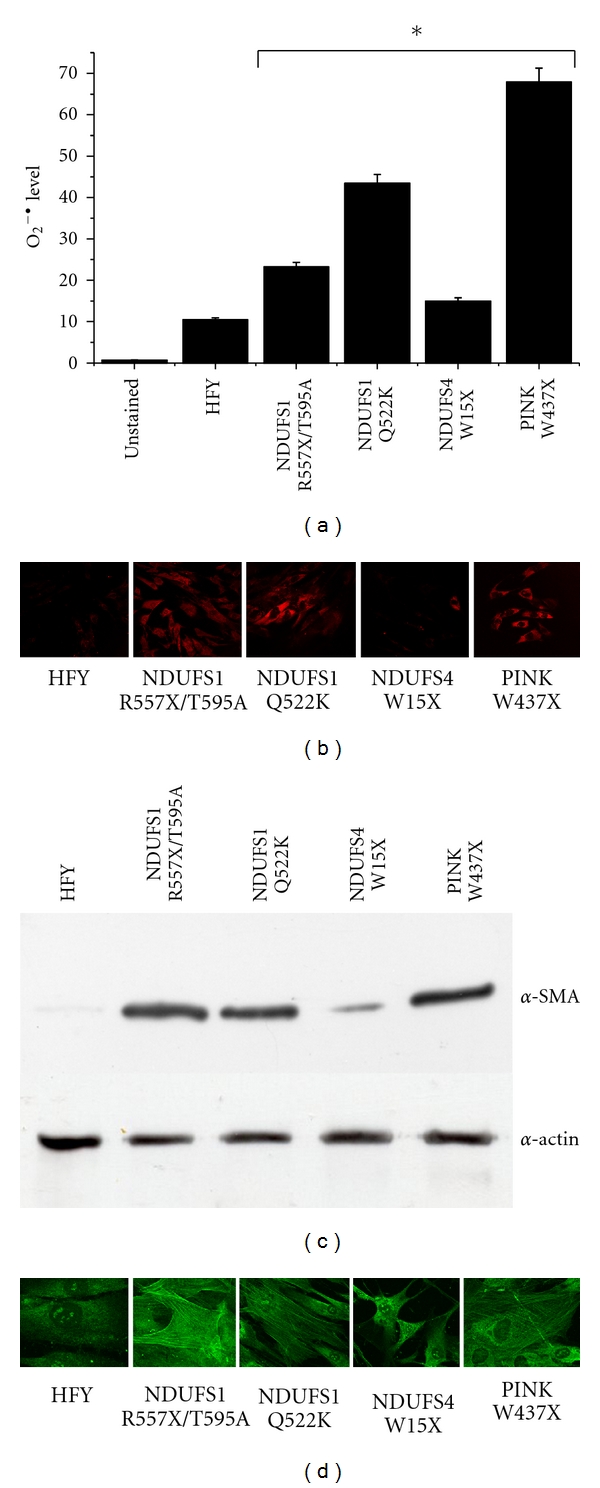
Oxygen superoxide level and *α*-SMA in fibroblasts carrying mitochondrial dysfunctions of complex I. (a) Flowcytometer analysis of O_2_^•−^ level. Control fibroblasts (HFY) and fibroblasts carrying mutations in *NDUFS1* gene (Q522K and R557X/T595A), in *NDUFS4* gene (W15X), and in the nuclear *PINK* gene (W437X) were cultured for 48 hours in low glucose medium and then incubated with 5 mM Mitosox for 10 minutes at 37°C for detection of oxygen superoxide. A flowcytometer analysis is then performed. The results are representative of five experiments with similar results. *P < 0.005 mutated fibroblasts versus control fibroblasts. (b) Fibroblasts seeded on glass coverslips are treated as in (a) and a confocal microscopy analysis is performed. (c) Analysis of *α*-SMA expression in control fibroblasts (HFY) and fibroblasts carrying mutations. Lysates of cells were subjected to *α*-SMA immunoblot analysis. An antiactin immunoblot was performed for normalization. (d) Analysis of *α*-SMA expression in control fibroblasts (HFY) and fibroblasts carrying mutations seeded on glass coverslips by confocal microscopy analysis.

We first detected the superoxide ion production by flow cytometer analysis and confocal microscopy analysis using Mitosox as a redox-sensitive probe. As shown in Figures 1(a) and 1(b), mutations in *NDUFS1* genes and in *PINK* gene are associated with superoxide ion accumulation while *NDUFS4* gene mutation affects only marginally ROS production in agreement with previous data [12]. Recently, it has been demonstrated that the oxidative stress in the tumour stroma promotes the conversion of fibroblasts into myofibroblasts, a contractile and secretory form of fibroblasts [7]. To this purpose we analysed whether also ROS produced by mitochondrial disfunctions could affect the differentiation process of fibroblasts. We analysed the expression level of *α*-SMA (*α*-smooth muscle actin), a typical marker of myofibroblast differentiation, by western blot and confocal microscopy analyses (Figures 1(c) and 1(d)). The level of *α*-SMA in control and mutated fibroblasts correlates with ROS production, in particular, in fibroblasts carrying mutations in the *NDUFS1* gene (Q522K and R557X/T595A) and in the nuclear *PINK* gene (W437X) *α*-SMA is organized in stress fibers, thereby conferring to the differentiated myofibroblasts a strong contractile activity. Altogether, these data show that ROS produced by fibroblasts carrying mitochondrial genetic disfunctions correlates with their activation. 

### 2.2. Mitochondrial ROS Production Induces an Increase in the Migration and Invasion Abilities of Myofibroblasts

**Figure 2:**
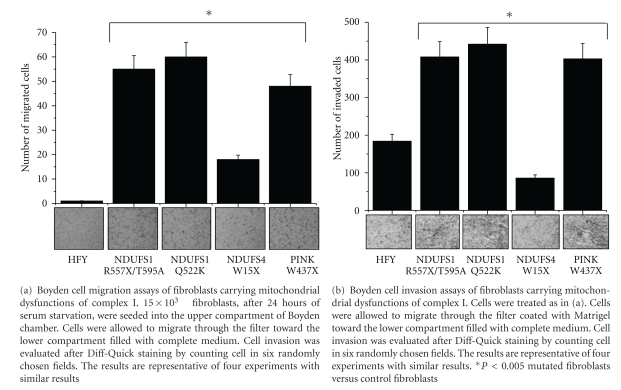
Mutations in *NDUFS1 *genes (Q522K and R557X/T595A) and in the nuclear *PINK* gene (W437X) increase fibroblasts migration and invasion.

During the conversion of fibroblasts into myofibroblasts, these cells acquire clear contractile and motile properties. To investigate whether fibroblasts carrying mitochondrial disfunctions and showing increased ROS level have modified their behaviour, we evaluated the migration and invasion abilities of these cells by Boyden assays. As shown in Figures 2(a) and 2(b), mutations in *NDUFS1* gene (Q522K and R557X/T595A) and in the nuclear *PINK* gene (W437X) increase both migration and invasion of fibroblasts while mutations in *NDUFS4* gene do not affect the contractile properties of cells, in agreement with their ROS production and *α*-SMA expression. Hence, we proposed that high mitochondrial generation of ROS converts fibroblasts into myofibroblasts, their activated form, causing an increase in the invasive and migratory abilities of these cells.

### 2.3. Hypoxic ROS Production in Mutated Fibroblasts Is Associated with HIF-1*α* Stabilization and Growth Factor Expression

**Figure 3:**
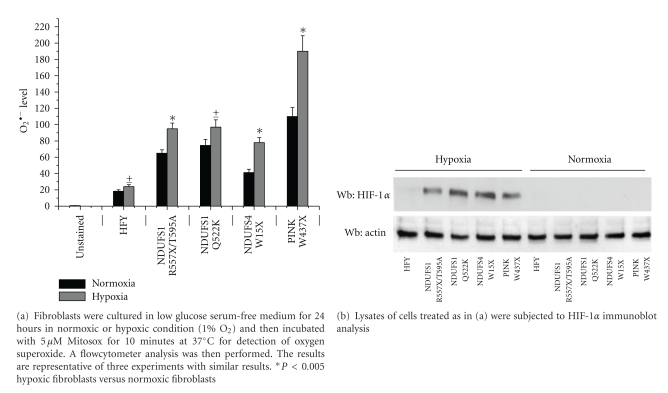
Mutated fibroblasts induce HIF-1*α* stabilization in hypoxic conditions.

**Figure 4:**
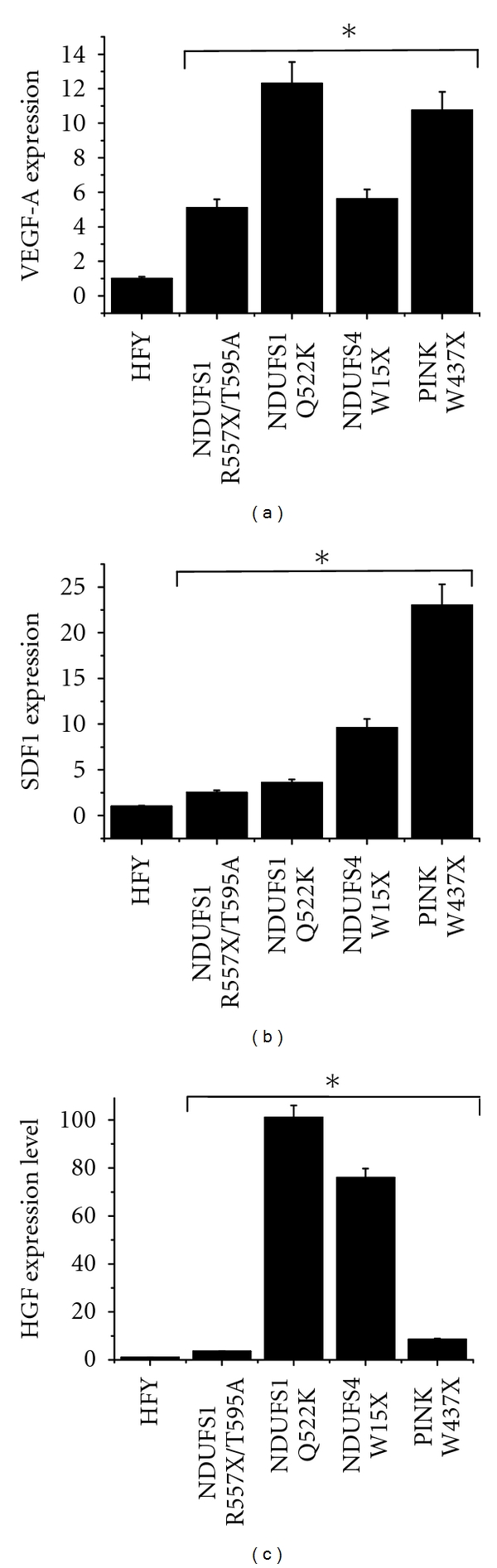
mRNA levels of VEGF-A, SDF-1, and HGF secreted by fibroblasts cultured in hypoxic condition. (a–c) Quantitative real-time reverse transcription PCR of RNA extracted from fibroblasts cultured for 24 hours in low glucose serum-free medium in hypoxic conditions using primers for human *VEGF-A *(a)*, SDF-1* (b), and* HGF *(c) and *GAPDH* gene. Results were normalized first to GADPH expression levels and then displayed relative to level in HFY cells. Data are representative of three independent experiments. *P < 0.005 mutated fibroblasts versus control fibroblasts.

Hypoxic conditions are able to induce a deregulation in mitochondrial ROS production, which control a variety of hypoxic responses, including the activation of HIF-1*α* transcription factor [14–16]. To this end, we analysed ROS production after culturing fibroblasts under hypoxic conditions (1% O_2_). As shown in Figure 3(a), genetic mitochondrial disfunctions result in increased ROS production in hypoxic conditions. This effect is mainly evident for fibroblasts carrying mutations in nuclear *PINK1* gene. Noticeable, also fibroblasts mutated in *NDUFS4* gene show high level of ROS in hypoxic conditions. In agreement, in hypoxic conditions, all mutated fibroblasts show an increase of HIF-1*α* level (Figure 3(b)). Previous results from other laboratories indicated that the activated stroma secretes large amounts of VEGF-A (vascular endothelial growth factor-A), SDF1 (stromal cell-derived factor-1) and HGF (hepatocyte growth factor) leading to a significant increase in the invasive capacity of surrounding tumor cells [17–19]. In order to verify whether the increased ROS production in mutated fibroblasts correlates with a raise of these soluble growth factors and cytokines, we performed a real-time PCR analysis to quantify VEGF-A, SDF1, and HGF transcripts. As shown in Figures 4(a) and 4(c) mutated fibroblasts cultured in hypoxic conditions have higher level of transcripts for VEGF-A, SDF1, and HGF, acknowledged factors for the modulation of the response of tumour cells to activated fibroblasts. 

### 2.4. The Conditioned Media of Mutated Fibroblasts Promotes Melanoma Cells Invasiveness

**Figure 5:**
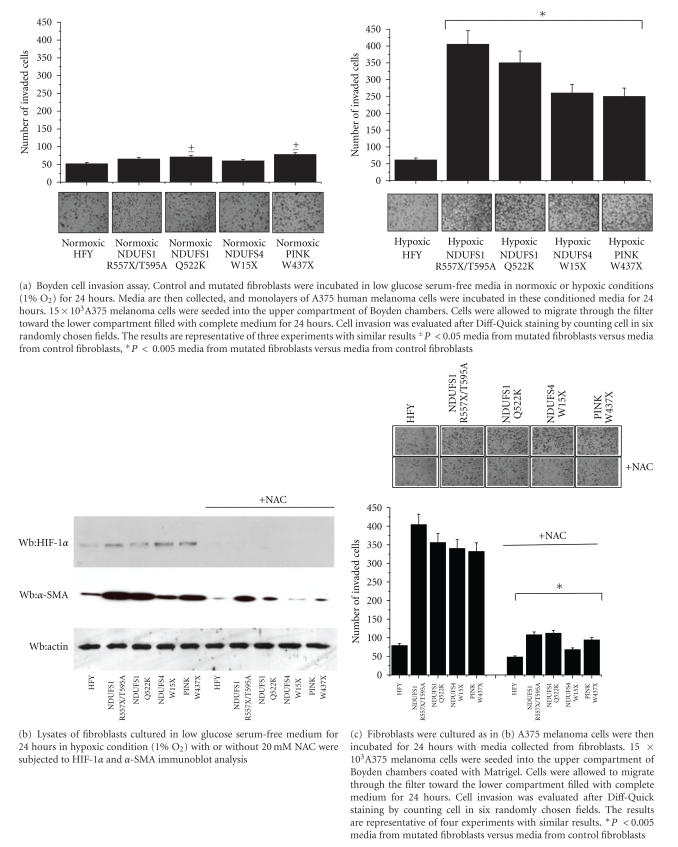
Conditioned media from mutated fibroblasts promotes melanoma cells invasiveness; NAC treatment blocks HIF-1*α* stabilization and reverts the increase of melanoma cells invasiveness.

Recently, it has been demonstrated that high levels of mitochondrial ROS produced by cancer cells are linked to enhanced metastatic potential [1]. To this end we decided to investigate whether mitochondrial ROS derived from stromal components could, as well, influence the behavior of tumor cells. Thus, we analysed the ability of media from mutated fibroblasts cultured under hypoxic conditions to promote metastatic potential of cancer cells, using A375 cells, derived from human primary melanoma. As shown in Figure 5(a), left, while there are no differences in A375 human melanoma cells invasiveness when cultured with media from fibroblasts grown in normoxic condition, media from mutated fibroblasts cultured in hypoxic conditions cause an increase in A375 human melanoma cells invasiveness as examined by Boyden cell invasion assay (Figure 5(a), right).

To verify that the effect exerted by media from mutated fibroblasts on the invasiveness of melanoma cells are effectively due to their ROS production and HIF-1*α* stabilization, we analyzed HIF-1*α* level and melanoma cell invasion in the presence of NAC (N-acetyl cysteine), a ROS scavenger. As shown in Figures 5(b) and 5(c), NAC treatment blocks HIF-1*α* accumulation and reverts the increase of invasiveness of A375 treated with media from mutated fibroblasts cultured in hypoxic conditions. Hence, this evidence underlines the involvement of mitochondrial ROS production in regulating the aggressiveness of melanoma cells, likely modulating the delivery of key cytokines able to affect cancer cell invasiveness.

## 3. Discussion

Data reported in this study clearly underscore the central role of mitochondrial ROS in the transdifferentiation of fibroblasts and in the stimulation of a pro-invasive phenotype of melanoma tumour cells.

Recent data unlighted that tumour microenvironment has a key role for the development of a variety of cancers promoting both tumour growth and metastatic dissemination. CAFs are the most common type of cells found in the reactive stroma of several human carcinomas. These activated fibroblasts express *α*-SMA, leading to the term “myofibroblasts,” influence ECM turnover synthesizing both components of ECM itself and ECM-degrading enzymes, release a large amount of cytokines affecting cancer cells progress towards an aggressive phenotype. The origin of myofibroblasts are not fully understood. In culture the transdifferentiation of fibroblasts to myofibroblasts can be achieved by treatment with TGF*β* (transforming growth factor *β*) [20], suggesting that similar pathways might be responsible for generation of myofibroblasts in tumours. Besides, emerging evidence indicates that also EMT, involving normal epithelial cells adjacent to the tumour, is a source of myofibroblasts in both fibrosis and cancer [21]. Furthermore, CAFs may arise directly from carcinoma cells through EMT [3, 7], allowing cancer cells to adopt a mesenchymal cell phenotype, with enhanced migratory capacity and invasiveness [22]. Indeed, mainly in breast cancers, have been reported CAF somatic mutations in *TP53* and *PTEN*, as well as gene copy number alteration at other loci in tumour stroma, [23, 24]. Actually, more recently, Radisky et al. [25], demonstrated that treatment of mouse mammary epithelial cells with MMP3 cause EMT through a pathways involving elevated ROS production and increased levels of Rac1b. The ROS increase in cells treated with MMP-3 was caused by mitochondrial activation and was found to be required for the induction of the mesenchymal vimentin as well as other myofibroblast genes. Thus, a new role for ROS in tumour progression is emerging, in addition to their well-known action in the oxidative damage to DNA. We have recently demonstrated that CAFs secrete MMPs which in turn stimulate ROS production in prostate carcinoma cells via a Rac1b/Cox2/HIF-1*α* pathway, finally leading to tumour growth and metastatic spreading [9]. Furthermore, Toullec et al., using a model of *junD*-deficient fibroblasts, demonstrated that the constitutive oxidative stress generated by inactivation of the *junD*-gene promotes the conversion of fibroblasts into myofibroblasts in a HIF1*α* and CXCL12-dependent pathway [19].

Mitochondria are the major site of ROS generation, which occurs mainly at complexes I and III of the respiratory chain. It is well known that mitochondrial disfunctions and increased ROS levels are present in many cancer cells. Pelicano et al. evidentiated a more invasive behavior of breast cancer cells in which oxidative stress is induced by inhibition of the electron transport complex I with rotenone [26]. A correlation between mutations in mitochondrial genes of complex I and cancer has been observed in various laboratories [1, 27, 28]. Recently, the group of G. Romeo reported, in the thyroid oncocytic cell line XTC.UC1, a dramatically decreased activity of complex I and III associated with ROS increase. Indeed, these defects are due to two mtDNA mutations: a frameshift mutation in the gene encoding ND1 subunit of complex I and a missense substitution in the cytochrome *b* gene, which affects the catalytic site involved in the electron transfer of complex III [29, 30]. Furthermore, mitochondria dysfunctions in a tumour cell line contribute to tumour progression by enhancing the metastatic potential of tumour cells [1]. Indeed, Ishikawa et al. demonstrated, by replacing the endogenous mtDNA in a mouse tumour cell line that was poorly metastatic with mtDNA from a cell line that was highly metastatic and viceversa, that the recipient tumour cells acquired the metastatic potential of the transferred mtDNA. Really, mtDNA containing mutations in the *ND6* (NADH dehydrogenase subunit 6) gene and hence a deficiency in respiratory complex I activity triggers an increase of ROS and higher expression levels of two genes associated with neoangiogenesis, namely, *HIF-1*α** and *VEGF*. As already mentioned, mitochondrial alterations in cancer cells have been intensively studied to understand their role in tumour development and progression [31, 32], but, at least to our knowledge, this manuscript, for the first time, evidenced that mitochondrial dysfunctions in stromal cells affect the invasiveness of cancer cells. Indeed, our data show that ROS produced by mitochondrial dysfunction affect not only the migratory and invasive abilities of fibroblasts themselves but also the aggressiveness of melanoma cancer cells. In order to investigate the involvement of mitochondrial ROS derived from stromal cells in modulating the invasiveness of tumour cells, we used fibroblasts producing elevated mitochondrial ROS due to defects in NADH: ubiquinone oxidoreductase, or complex I. Dysfunctions of this complex cover more than 30% of hereditary mitochondrial encephalopathies [33–35]; furthermore, complex I defects have been also found in familiar Parkinson disease [11, 13], hereditary spastic paraplegia [36], Friedreich ataxia [37], and aging [38, 39]. We used fibroblasts from a baby diagnosed for Leigh syndrome (NDUFS4 W15X), from a baby diagnosed for leukodystrophy (NDUFS1 Q522K), from a child with complex I deficit (NDUFS1 R557X/T595A), and from a patient with familiar Parkinsons disease (PINK W437X) in which mutations in the *ND5 *and *ND6 *mitochondrial genes of complex I coexist with mutation in the nuclear phosphatase and tensin homolog- (PTEN-) induced serine/threonine putative kinase-1 (*PINK-1*) gene. We showed that in normoxic conditions all mutations present in these fibroblasts, with the exception of the mutation in the gene *NDUFS4*, produce an increase in ROS level, which correlates with the levels of *α*-SMA and hence with the degree of differentiation towards the myofibroblast-activated form. This differentiation represents also a key event during wound healing and tissue repair [4]. The deregulation of normal healing and continued exposure to chronic injury results in tissue fibrosis, massive deposition of ECM, scar formation, and organ failure. Indeed, oxidative stress, caused by increase in ROS is closely associated with fibrosis [40] and inhibitors of ROS have shown promise in clinical trials targeting this disease [41–43]. Herein, we demonstrate that mutated fibroblasts have a great ability to increase their motility and invasiveness. Overall, these findings indicate that increased mitochondrial ROS induce the transdifferentiation of fibroblasts to their activated form suggesting a possible involvement of these mutated fibroblast also in fibrogenic events.

The inappropriate induction of myofibroblasts that leads to organ fibrosis greatly enhances the risk of subsequent cancer development, by creating a stimulating microenvironment for epithelial tumor cells. Really, we show that exposure of melanoma cells to media of fibroblasts with mitochondrial dysfunctions cultured in hypoxic conditions promotes invasiveness of tumour cells. It is well known that in hypoxic condition, the low oxygen tension increases the generation of mitochondrial ROS that prevent hydroxylation of HIF1*α* protein, thus resulting in stabilization and activation of its transcriptional activity [14, 15, 44]. Recently, for instance, Klimova et al. demonstrated that ROS generated by mitochondrial complex III are required for the hypoxic activation of HIF1*α* [16]. Furthermore, Brunelle et al. showed that fibroblasts from a patient with Leigh's syndrome, which display residual levels of electron transport activity, stabilize HIF1*α* during hypoxia [45]. Really, we evidentiated in hypoxic conditions an extra ROS production in fibroblasts carrying mutations in mitochondrial complex I; this increase in ROS production is also evident in fibroblasts mutated in the *NDUFS4 *gene that, conversely, have a low level of ROS in normoxic condition. The extra ROS production in hypoxic condition is associated with HIF1*α* stabilization. This stabilization is really dependent on mitochondrial mutations since it is absent in control fibroblasts and finally leads to the transcriptional activation of genes that allow cells to adapt to and survive in the hypoxic environment. We believe that this extra ROS production acts synergistically with the hypoxic condition in the promotion of a pro-invasive behavior of melanoma cancer cells. Neutralizing this extra ROS production with antioxidants allows the degradation of HIF1*α* and abolishes the aggressive behavior of melanoma cancer cells. Altogether we evidentiated that mitochondrial ROS produced by complex I defects of stromal components, namely, activated fibroblasts, are key molecules able to modulate the behavior of surrounding cancer cells, increasing their aggressiveness.

These data underline once again the close loop between tumor cells and stromal counterparts. It is well known that activated fibroblasts influence, through the secretion of soluble factors, the adhesive and migratory properties of cancer cells, which then in turn release cytokines influencing the behavior of stromal cells. Thus, to deeply explore the effect of mutated fibroblasts on surrounding tumour cells we investigated the transcriptional levels of some HIF1*α* target genes in fibroblasts cultured in hypoxic conditions. We observed an increase in VEGF-A, HGF, and SDF1 transcripts. These data are in keeping with those obtained by Orimo et al. [46], demonstrating that the coinjection of tumour cells with CAFs into nude mice generates larger xenografts with respect to those generated with normal fibroblasts. This event correlates with an increase in both cancer-cell proliferation and angiogenesis through the SDF-1 secretion. In agreement, Toullec et al. demonstrated that the same cytokine causes the conversion of fibroblasts into highly migrating myofibroblasts and subsequently promotes migration and dissemination of neoplastic cells [19]. Furthermore, Cat et al. showed that myofibroblasts secrete large amounts of HGF and VEGF resulting in a significant increase in the invasive capacity of surrounding tumor cells [17]. Besides, clinical studies show that the abundance of stromal myofibroblast is associated with disease recurrence, as shown for human colorectal cancers [47]. Really, our data, showing an increase in fibroblast secreted VEGF-A, HGF, and SDF1, are in keeping with others emphasizing that tumour dissemination could be facilitated by the myofibroblastic component of the stroma through the secretion of invasion associated-secreted factors.

Altogether our findings suggest a possible role of ROS production, due to mitochondrial complex I dysfunctions of stroma, in fibroblast activation as well as in cancer progression and invasion.

## 4. Materials and Methods

### 4.1. Materials

Unless specified, all reagents were obtained from Sigma. Antibodies anti-HIF-1*α* were from BD Transduction Laboratories; antibodies anti-*α*-SMA were from Sigma; antibodies anti-actin were from Santa Cruz Biotechnology; MitoSOX Red mitochondrial superoxide indicator was from Molecular Probes.

### 4.2. Cell Culture

Control fibroblasts (neonatal human dermal fibroblasts) were from Cambrex. Fibroblasts carrying mitochondrial mutations in the nuclear *NDUFS4* gene*-*W15X and in the *NDUFS1* gene-Q522K (from M. Zeviani, C. Besta Neurological Institute Foundation, Milan) were cultivated and characterized by S. Scacco. Fibroblasts carrying mutation in the nuclear *PINK1* gene-W437X (from G. De Michele, Department of Neurological Sciences, Federico II University, Naples) were cultivated and characterized by A. M. Sardanelli. Fibroblasts carrying mitochondrial mutations in the nuclear *NDUFS1* gene (NDUFS1 R557X/T595A) were a generous gift from Fondazione Giuseppe Tomasello O.N.L.U.S. A375 human melanoma cells were from ATCC. Cells were cultured in DMEM supplemented with 10% fetal bovine serum and maintained in 5% CO_2_ humidified atmosphere.

### 4.3. Mitochondrial Superoxide Detection

25 × 10^3^ cells were cultured for 48 hours in low glucose DMEM medium (5 mM glucose). Cells were then incubated for 10 minutes at 37°C with 5 *μ*M MitoSox in PBS. Cells are trypsinized, centrifuged, washed with PBS, and resuspended in 300 *μ*L PBS. A flowcytometer analysis was then performed (MitoSox excitation/emission: 510/580 nm). For the confocal microscope analysis of O_2_^•−^ level, cells were seeded onto coverslips, incubated for 10 minutes at 37°C with 5 *μ*M MitoSox in PBS, washed with PBS, and analysed with a laser scanning confocal microscope (model LEICA TCS SP2 with Acusto-Optic Beam Splitter) equipped with a five-lines Ar laser and two He/Ne lasers (lines 543 and 633 nm).

### 4.4. Western Blot Analysis

1 × 10^6^ cells were lysed for 20 minutes on ice in 500 *μ*L of complete radioimmunoprecipitation assay (RIPA) lysis buffer (50 mM Tris-HCl (pH 7.5), 150 mM NaCl, 1% NP40, 2 mM EGTA, 1 mM sodium orthovanadate, 1 mM phenylmethylsulfonyl fluoride, 10 *μ*g/mL aprotinin, 10 *μ*g/mL leupeptin). Lysates were clarified by centrifuging, separated by SDS-PAGE, and transferred onto nitrocellulose. The immunoblots were incubated in 3% bovine serum albumin, 10 mM Tris-HCl (pH 7.5), 1 mM EDTA, and 0.1% Tween 20 for 1 hour at room temperature and were probed first with specific antibodies and then with secondary antibodies.

### 4.5. Immunohistochemistry

Fibroblasts were seeded onto coverslips, washed with PBS, and fixed in 3% paraformaldehyde for 20 minutes at 4°C. Fixed cells were permeabilized with three washes with TBST (50 mM Tris/HCl pH 7.4, 150 mM NaCl, 0.1% Triton X-100) and then blocked with 5.5% horse serum in TBST for 1 hour at room temperature. Cells were incubated with primary antibody, 1 : 100 dilution in TBS (50 mM Tris/HCl pH 7.4, 150 mM NaCl) containing 3% BSA overnight at 4°C. After extensive washes in TBST, cells were incubated with secondary antibodies for 1 hour at room temperature, washed, and mounted with glycerol plastine. Finally, cells were observed under a laser scanning confocal microscope (model LEICA TCS SP2 with Acusto-Optic Beam Splitter) equipped with a five-lines Ar laser and two He/Ne lasers (lines 543 and 633 nm).

### 4.6. Preparation of Conditioned Media

Conditioned media were obtained from fibroblasts as follow: fibroblasts were incubated in low glucose (5 mM glucose) serum-free media in normoxic or hypoxic conditions (1% O_2_) for 24 hours. Media are then collected and monolayers of A375 human melanoma cells were incubated in these conditioned media for 24 hours.

### 4.7. In Vitro Boyden Migration and Invasion Assay

Fibroblasts were serum starved for 24 hours and then 15 × 10^3^ cells were seeded onto Boyden chamber (8 mm pore size, 6.5 mm diameter) for the migration assay. For the invasion assay Boyden chambers are precoated with Matrigel (12.5 *μ*g Matrigel/filter). In the lower chamber, complete medium was added as chemoattractant. Following 24 hours of incubation, the inserts were removed and the noninvading cells on the upper surface were removed with a cotton swab. The filters were then stained using the Diff-Quik kit (BD Biosciences) and photographs of randomly chosen fields are taken.

### 4.8. Real-Time PCR

Total RNA from fibroblasts was extracted using RNeasy (Qiagen) according to the manufacturer instructions. Strands of cDNA were synthesized using a high-capacity cDNA reverse transcription kit (Applied Biosystem) using 1 *μ*g of total RNA. For quantification of VEGF-A, SDF-1, and HGF mRNA, real-time PCR, using Power SYBR green dye (Applied Biosystem) was done on a 7500 fast real-time PCR system (Applied Biosystem). The primers for VEGF-A were 5′TTCTGCTGTCTTGGGTGCAT-3′ (forward) and 5′TGTCCACCAGGGTCTCGATT-3′ (reverse). The primers for SDF-1 were 5′GTGTCACTGGCGACACGTAG-3′ (forward) and 5′TCCCATCCCACAGAGAGAAG-3′ (reverse). The primers for HGF were 5′CATCAAATGTCAGCCCTGGAGTT-3′ (forward) and 5′CCTGTAGGTCTTTACCCCGATAGC-3′ (reverse). Data are normalised to those obtained with glyceraldehyde-3-phosphate dehydrogenase primers. Results (mean ± SD) are the mean of three different experiments.

## Abbreviations

*α*-SMA: *α*-smooth muscle actin
CAF: Cancer associated fibroblast
ECM: Extracellular matrix
EMT: Ephitelial mesenchymal transition
HGF: Hepatocyte growth factor
HIF-1*α*: Hypoxia inducible factor-1*α*
MMP: Matrix metalloproteinase
NAC: N-acetyl cysteine
ROS: Reactive oxygen species
SDF1: Stromal cell-derived factor-1
VEGF: Vascular endothelial growth factor.
